# Comparison of Radioimmuno and Carbon Nanotube Field-Effect Transistor Assays for Measuring Insulin-Like Growth Factor-1 in a Preclinical Model of Human Breast Cancer

**DOI:** 10.1186/1477-3155-9-36

**Published:** 2011-09-02

**Authors:** Laundette P Jones, Steingrimur Stefansson, Man S Kim, Saeyoung N Ahn

**Affiliations:** 1 Department of Pharmacology and Experimental Therapeutics, University of Maryland School of Medicine, 655 West Baltimore St. BRB-400-2 Baltimore, Maryland 21201 USA; 2 Fuzbien Technology Institute 9700 Great Seneca Hwy, Suite 302, Rockville, MD, 20850, USA

**Keywords:** Biomarker, BRCA1, Carbon Nanotube, IGF-1, mouse

## Abstract

**Background:**

To realize the promise of personalized medicine, diagnostic instruments used for detecting and measuring biomarkers must become smaller, faster and less expensive. Although most techniques used currently to detect biomarkers are sensitive and specific, many suffer from several disadvantages including their complexity, high cost and long turnaround time. One strategy to overcome these problems is to exploit carbon nanotube (CNT) based biosensors, which are sensitive, use inexpensive disposable components and can be easily adapted to current assay protocols. In this study we investigated the applicability of using a CNT field-effect transistor (CNT-FET) as a diagnostic instrument for measuring cancer biomarkers in serum using a mouse model of *Breast Cancer Susceptibility 1*-related breast cancer. Insulin like growth factor-1 (IGF-1) was chosen because it is highly relevant in breast cancer and because measuring serum IGF-1 levels by conventional methods is complicated due to specific IGF-1 serum binding proteins.

**Findings:**

Our results show that there is good correlation between the two platforms with respect to detecting serum IGF-1. In fact, the CNT-FETs required only one antibody, gave real-time results and required approximately 100-fold less mouse serum than the radioimmunoassay.

**Conclusions:**

Both IGF-1 radioimmuno and CNT-FET assays gave comparable results. Indeed, the CNT-FET assay was simpler and faster than the radioimmunoassay. Additionally, the low serum sample required by CNT-FETs can be especially advantageous for studies constricted by limited amount of human clinical samples and for mouse studies, since animals often need to be sacrificed to obtain enough serum for biomarker evaluation.

## Findings

Insulin-like growth factor-1 (IGF-1) is a pleiotropic 70 amino acid peptide produced mainly by the liver. It is a potent mitogen and survival factor for many cell types including smooth muscle, epithelial and interstitial cells and is vital for normal development and cell differentiation. Conversely, it also has a role in abnormal physiology such as mammary carcinogenesis and tumor growth [1]. Circulating levels of IGF-1 are positively associated with increased breast cancer risk in pre- and postmenopausal women, particularly for estrogen-receptor positive tumors [2-4]. Moreover, the IGF-1/IGF-1 receptor axis has also been shown to be involved in the increased risk of early-onset breast cancers in women with mutations in the *Breast Cancer Susceptibility gene *(*BRCA1*). [5-7]. Germline mutations in *BRCA1 *have been detected in approximately half of human familial breast cancer cases [8,9].

In order to gain insights into the downstream factors involved in human BRCA1-associated breast cancers, a mouse model was developed with a conditional Brca1 gene deletion [10]. This mouse model demonstrates a pattern of progressive adenocarcinoma with similar genetic changes and pathophysiology as seen in human breast cancers associated with BRCA1-mutations [11,12]. Additionally, as in human BRCA1-associated breast cancer, increased expression of several components of the IGF axis is seen in liver, normal mammary tissue and mammary tumors of these mice along with increased levels of IGF-1 in serum [13].

Currently, breast self-exams and mammograms are the predominant methods used to detect breast cancer in its early stages. Unfortunately, blood tests for breast cancer biomarkers are not yet a routine diagnostic procedure as for many other cancers, but many studies have shown that IGFs, IGF binding proteins (IGFBPs) and IGF receptors are good candidates for breast cancer markers because they are strong prognostic factors for breast cancer outcomes [4,14-16]. Because of the need for fast and inexpensive diagnostic tools to detect risk factors associated with breast cancers and other malignancies, we investigated the possibility of using a carbon nanotube field-effect transistor (CNT-FET) to measure serum IGF-1 levels in the Brca1-associated mouse model of human breast cancer. This assay was compared to a radioimmunoassay (RIA) method that is performed by clinical laboratories.

**Figure 1:**
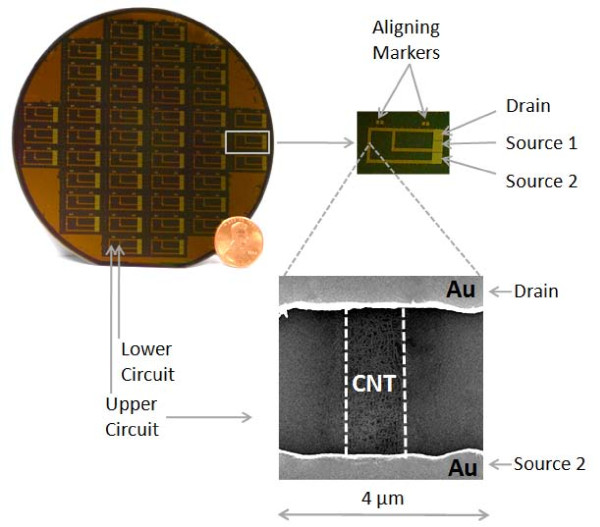
**Current Fuzbien Technology Institute (FTI) CNT-FET**. Each 4'' silica semiconductor wafer has forty six 0.45''x 0.15''cells containing 2 independent CNT circuits (upper and lower). An enlarged view of a cell indicating the contact surfaces of the source and the drain is shown to the right of the wafer. Gate voltage is applied at the back of the wafer. Also indicated are the aligning markers for the lithography printing system. Below the cell is a SEM of a circuit showing the CNT's sandwiched between the gold source and drain electrodes.

CNTs are two-dimensional graphene sheets forged into elongated tubes which display unique physical attributes, such as high tensile strength and excellent electrical conductivity, which makes them attractive for use in nano-scale biodetectors. Additionally, CNT-based biodetectors are versatile and can use either antibody, aptamer or avidin-biotin based capture [17-23]. The CNT-FET wafer design used in this study is shown in Figure 1 and was developed by Fuzbien Technology Institute (FTI, Rockville, MD). It is a semiconductor element that has three terminals; a source, drain and gate electrode, which is a configuration similar to that of conventional silicon metal-oxide-semiconductor field-effect transistors (MOS-FET). The wafer has 92 independent CNT-FET circuits that can handle sample volumes between 1-5 μl. The CNT-FET assay procedure is similar to immunodetection methods, such as an Enzyme-Linked Immunosorbent Assay (ELISA), in that an immobilized antibody is first used to capture the ligand. Unlike ELISA, which requires a labeled secondary antibody to generate a detectable signal, the CNT-FET detects the electrical properties of the bound ligand. When a charged ligand is in close proximity to a CNT carrying a current, the impedance (resistance) either increases or decreases. This change in impedance upon IGF-1 binding to the immobilized anti-IGF-1 antibody happens in real time. The impedance from the CNT-FET wafer is fed to a laptop containing a data acquisition program, which display the results with resolution down to 10^-10 ^Amp and resistance up to 10^9 ^Ohm.

Using a sandwich ELISA format to measure serum IGF-1 presents a problem because more than 95% of IGF-1 in circulation is present in high molecular weight complexes, mostly with IGFBP-3 [17]. There are six IGFBPs in circulation that can bind IGFs with high affinity and interfere with antibody-based detection, especially when a 2 antibody sandwich format is used. Therefore the IGF-1 RIA kit used in this study includes a denaturing step to dissociate the IGF-1: IGFBP-3 complex. First, a mild denaturing step employing a low pH buffer is used to dissociate the IGF-1-IGFBP complex. Then the serum sample is returned to physiological pH in the presence of excess IGF-2 provided in the kit. The excess IGF-2 saturates the refolded IGFBPs present in the sample, leaving the IGF-1 free to bind the antibodies. In contrast, the CNT-FET required only one antibody and no pre-treatment of the serum sample was needed. Since the CNT-FET is only sensitive to the electrical properties of a bound antigen, denaturing and dissociating a specific protein-antigen complex is not required to achieve a signal. Additionally, the CNT-FET assays used 2-5 μl of 1:10-1:50 diluted mouse serum per circuit, whereas the RIA required at least 50-100 μl of undiluted mouse serum.

**Figure 2:**
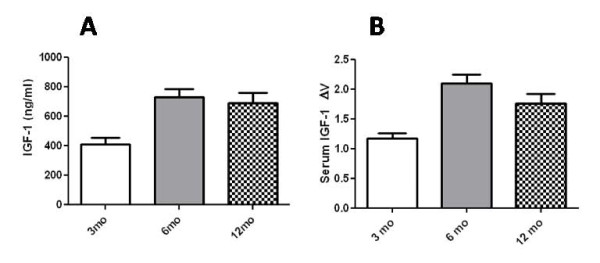
**Measurement of IGF-1 in mouse serum**. Mouse serum IGF-1 was measured using (A) radioimmuno assay and (B) CNT-FET. The impedance value for each IGF-1 measurement was normalized to the corresponding PBS baseline value. Both assays show an increase in the serum IGF-1 at 6 months compared to 3 months. Between After 6 and 12 months there is not a significant difference in the IGF levels between the age groups. Statistical differences among groups were analyzed using GraphPad Prism *t *tests (GraphPad Software, San Diego, CA). The values represent the average of ≥ 4 measurements ± standard error of the mean (SEM). Significance of *P *≤ 0.05 is indicated with an asterisk.

Both the RIA and CNT-FET assay gave comparable results using the same mouse plasma samples, namely a statistically significant increase in serum IGF-1 from Brca1^f/f; MMTV-Cre ^mice between 3 to 6 months of age with no further increase between 6-12 months (Figure 2, **panels A and B**). No significant difference in IGF-1 serum levels were seen in age matched CL57Bl/6 mice (data not shown). These data complement the studies of Shukla et al [13] that show an increase in serum IGF-1 levels after 3 months in a mouse model that is p53^+/- ^and Brca1 ^f/f; MMTV-Cre^, compared to normal CL57Bl/6 controls [13]. But at this point it is unclear whether alterations in Brca1, p53, or both are responsible for the increased IGF-1 levels. Another possible explanation of why we did not see a further increase in IGF-1 serum levels beyond 6 months of age is that none of those Brca1^f/f; MMTV-Cre ^mice at that age developed mammary tumors.

We anticipate that CNT-based biosensors will ultimately provide a rapid clinical tool to accurately and inexpensively help define the therapeutic potential of candidate biomarkers, such as IGF-1 for the early detection of breast cancer. These studies are important since the breast cancers from populations of women at high risk have elevated levels of circulating IGF-1 (e.g. women with BRCA1 mutations) are more difficult to treat/manage due to their aggressive nature and because the patients are often not candidates for standard endocrine therapy [24-26]. Furthermore, the low sample volumes used for the CNT-FETs means that a small blood sample can be used to screen for multiple biomarkers. A standard 8 ml vacutainer can yield enough serum for approximately 5000 measurements. Apart from making blood testing faster and more accessible, the low volume used by the CNT-FETs can be especially important where sample volumes are limited as in the case of blood from premature infants, biopsy samples from cancer patients and cerebrospinal fluid from patients suffering from neurodegenerative diseases such as Alzheimer's and Parkinson disease.

### Transgenic Mice and Sample Preparation

Brca1 conditional knockout mice with two floxed Brca1 alleles (Brca1^f/f^) carrying the mouse mammary tumor virus (MMTV)-Cre recombinase gene (Brca1^f/f; MMTV-Cre^) were maintained on a C57Bl/6 genetic background [10]. These mice continue to express the normal splice variant of Brca1 that lacks exon 11 and develop chromosome abnormality and tumorigenesis at low frequency after a long period [10,11]. Specifically, approximately 25% of mice develop mammary adenocarcinomas by 12 month of age when both p53 alleles are intact but is increased significantly (37-80%) by p53 haploinsufficiency [10,11]. The presence of the floxed Brca1 alleles, of wild-type Brca1 alleles, and of MMTV-Cre was identified by performing DNA polymerase chain reactions (PCR) on tail bleeds using primers described previously [27,10]. The transgenic mice and wild type C57Bl/6 mice were maintained in temperature-controlled and light-controlled conditions in the University of Maryland, Baltimore animal facility and maintained in accordance with institutional guidelines approved by the University of Maryland, Baltimore Animal Care and Use Committee. To compare RIA and CNT-FET assays, Brca1^f/f; MMTV-Cre ^mice and wild type C57Bl/6 mice were euthanized at 3, 6 and 12 months of age to collect trunk blood. For histology, mammary tissue from 3, 6 and 12 month old mice was removed post mortem and formalin fixed for histology and stained with hematoxylin and eosin.

### IGF-1 Radioimmunoassay

Three to five female mice were used per group. Mouse IGF-I serum levels were measured using a RIA kit containing microplates coated with the capture antibody and a I^125 ^labeled detector antibody (Alpco Diagnostic, Salem, NH). The assay was performed according to manufacturer's instructions. Statistical differences among groups were analyzed using GraphPad Prism *t *tests (GraphPad Software, San Diego, CA). Data are presented as means ± S.E.M. Significance was assigned at *P *≤ 0.05.

### IGF-1 CNT-FET Assay

**Figure 3:**
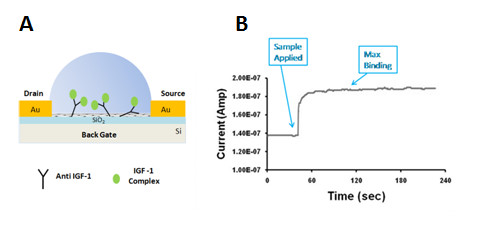
**A response of IGF-1 binding to the anti-IGF-1 antibody immobilized on CNT-FET**. (A) Experimental setup of the assay. (B) Real time binding of mouse serum IGF-1 to mouse anti-IGF-1 coated CNT-FET. Typical time of the assay from sample application to maximum binding is 30-90 sec.

Single-walled carbon nanotubes (SWNTs) were purchased from Carbon Nanotechnologies Inc. The SWNTs mixture used contains about 70% conducting nanotubes that have diameters between 0.7 to 1.4 nm and length between 20 to 80 nm. 92 sample-well CNT wafers were manufactured by NanoPlatform Inc., using standard photolithography and lift-off process. CNT's were then functionalized with pyrene butanoic acid succinimidyl ester as previously described [28]. An anti mouse IGF-1 was purchased from Abcam and diluted in PBS to 20 μg/ml. 5 μl of the antibody dilution was added to each CNT-FET circuit and incubated for 1 hr at RT followed by blocking with 0.001% BSA, washing with diH_2_O and drying with N_2 _gas. For the assays, a baseline impedance value for the circuit was obtained using PBS for 30 sec, after which 5 μl of purified recombinant mouse IGF-1 (eBioscience), diluted from 1-1000 ng/ml in PBS or mouse serum, diluted 1:10-1:50 in PBS, were added to the CNT-FET and change in impedance was measured for 3 min. The impedance value for each IGF-1 measurement was normalized to the corresponding PBS baseline value. Each sample was measured at least in quadruplicate using a fresh circuit for each measurement. A source/drain bias of 100 mV was maintained throughout the measurements of the electrical signal and the pulse width was 1 sec. The reference electrode is the back (bottom) side of the grounded wafer. A schematic of the experimental setup of the assay is depicted in Figure 3, panel A. The device uniformity was not optimized for entire wafers, but individual circuits used for the assays were carefully evaluated before the experiment. The selected CNT-FET circuits ranged typically between five and ten in on/off ratio. The electrical properties of the samples binding the CNT-FET were measured using a low current measurement system (LCM) by MediSourcePlus Inc. that makes electrical contact to the source and drain electrodes of the CNT-FET. The transfer characteristics of this circuit design were previously characterized for detection of prostate specific antigen [28]. Briefly, typical observed electronic transfer changes from 20 to 10 nano amperes before and after the antibody immobilization on the CNT-FET circuits when V_ds _and V_G _are 0.1 and -0.1 volt, respectively. With the IGF introduced on the circuit, the response in the electrical signal is typically in the range of 2 to 15% in the normalized units. A response of IGF-1 binding to the anti-IGF-1 antibody immobilized on our CNT-FET is shown in Figure 3B.

## List of Abbreviations

BRCA1: Breast Cancer Susceptibility gene; CNT: carbon nanotube; CNT-FET: carbon nanotube field-effect transistor; ELISA: Enzyme-Linked Immunosorbent Assay; IGF-1: Insulin like growth factor-1; IGFBP: IGF binding protein; LCM: low current measurement system; MMTV: mouse mammary tumor virus; RIA: radioimmunoassay; SWNT: Single-walled carbon nanotubes

## Competing interests

The authors wish to declare that SS, MK and SNA are with FTI which developed the CNT-FET wafers and instruments used in this study.

## Authors' contributions

SS and MSK performed the IGF-1 CNT-FET assays; LPJ coordinated the research effort and performed the IGF-1 radioimmuno assays. SNA developed the CNT-FET platform and reader. All authors have read and approved the final manuscript.
